# A Comparison of Brain-State Representations of Binary Neuroimaging Connectivity Data. Comment on Samantaray et al. Unique Brain Network Identification Number for Parkinson’s and Healthy Individuals Using Structural MRI. *Brain Sci.* 2023, *13*, 1297

**DOI:** 10.3390/brainsci14050422

**Published:** 2024-04-25

**Authors:** Josephine Noah Kelly, Bowen Fu, Zhenyu Li, Robert Emmett Kelly

**Affiliations:** 1 College of Engineering, Cornell University, Ithaca, NY 14850, USA; 2 College of Human Ecology, Cornell University, Ithaca, NY 14850, USA; bf357@cornell.edu (B.F.); zl859@cornell.edu (Z.L.); 3 Department of Psychiatry, Weill Cornell Medicine, White Plains, NY 10605, USA; rek2005@med.cornell.edu; 4 Department of Clinical Sciences, Lund University, 22100 Lund, Sweden

Samantaray et al. [1] introduced a new method for storing binary neuroimaging connectivity data with the goal of providing a compact, space-saving representation of brain-state connectivity, called the Unique Brain Network Identification Number (UBNIN). They utilized UBNINs to present binary connectivity data, an adjacency matrix, derived from structural brain MRI, which represented the strength of connections (edges) between areas of the brain, divided into 56 nodes. Fifty-six nodes correspond to 55 × 56/2 = 1540 edges and 2^1540^ possible unique combinations of edges. The authors demonstrated how the UBNIN could facilitate the storage of a unique combination of such an unwieldy number of edges. Here, we provide a comparison of UBNIN with three other, more conventional methods for storage of such brain-state data: binary, base 10, and base 16 (hexadecimal) representations. We compare method procedures and the amount of space required in print and in electronic storage to record these binary brain-state representations. Our focus here is not on how to derive these binary matrices, but on how to best encode and store them.

The UBNIN method, as presented in Samantaray et al.’s paper [1], is actually three related methods, which we call here the complete UBNIN (UBNIN-C), the rounded UBNIN (UBNIN-R), and the truncated UBNIN (UBNIN-T). UBNIN-C encodes all of the information in the adjacency matrix but can become too long to be managed easily. UBNIN-R is the value of UBNIN-C rounded to a given precision, which in the paper was 15 digits. UBNIN-T is the value derived by dropping the fractional portion of the number and keeping only the integer portion. For large matrices, UBNIN-R and UBNIN-T provide a more compact and manageable representation, at the cost of loss of information, as described below.

We begin by summarizing the complete UBNIN method, which is described in more detail by the authors. The binary data from the adjacency matrix are organized into columns that are represented by a base 10 number for each column (Figure 1). These numbers are sequentially added together after the partial sums are first divided by increasing powers of 2 in such a way that after each iteration, the integer part of the partial sum is the number from the last column added, and the fractional part contains the encoded information from all of the other numbers added previously (Figure 2). It works out this way because the quotient is always less than 1 after dividing by the increasing powers of 2. The final UBNIN-C contains an integer part that is the base 10 representation of the edges for the last column’s node. For example, consider a UBNIN derived from only the first four nodes shown in Figure 1. Following the left-hand flowchart of Figure 2 we see that UBNIN(1) = 0; UBNIN(2) = 1 + 0 = 1; UBNIN(3) = 3 + 0.5 × 1 = 3.5; and UBNIN(4) = 6 + 0.25 × 3.5 = 6.875. These are the UBNIN-C numbers shown in the second column of Table 1. The binary number corresponding to UBNIN(4) is formed by concatenating the numbers 1, 11, and 110 (reading from bottom to top in columns 2, 3, and 4 of the adjacency matrix in Figure 1). Conversion of this binary number, 111110, to base 10 and hexadecimal yields the numbers shown in Table 1, corresponding to the number of nodes = 4.

Decoding this UBNIN-C recreates the base 10 numbers for each column of the adjacency matrix by reversing the encoding process, multiplying the fractional part by the appropriate power of 2 in a cyclical process until all of the original numbers are found, in the integer parts of these decoded numbers. The last number found, corresponding to column 2, must either be a 0 or 1, with no fractional part, so this provides a check for errors in the fractional portion of the UBNIN-C value, or in the decoding process.

This potential for error checking means that UBNIN-Cs take up more space in print and in electronic data storage than conversion to a base 10 number, as demonstrated in Table 1 for the number of nodes from 2 to 10. The number of digits required for any base 10 representation (using digits 0–9) of numbers as high as 2*^p^* would be the smallest integer greater than *k* × *p*, where *k* = log_10_(2) ≅ 0.30 [2]. For hexadecimal representation, where each digit corresponds to a four-digit binary number, the number of digits is divided by 4. For 56 nodes, with 2^1540^ combinations, hexadecimal representation would be the most efficient in print, requiring 0.25 × 1540 = 385 digits. Base 10 would require 464 digits, and UBNIN-C, many more.

The exact number of digits for UBNIN-C can be calculated as follows. If we consider dividing by 2*^q^* in terms of the equivalent multiplication by the reciprocals (0.5, 0.25, 0.125, etc.), we see that the number of digits to the right of the decimal point for these reciprocals is equal to *q*. The UBNIN-C’s digits to the right of the decimal point grow by the number of digits to the right of the decimal point of the multiplied numbers, which means that the total number of digits to the right of the decimal point would be given by the series 1 + 2 + 3 … + *q* = *q* × (*q* + 1)/2. Thus, for 56 nodes, the number of UBNIN-C digits required would be 54 × 55/2 = 1485, in addition to up to 17 digits to the left of the decimal point, not much less than the 1540 digits required for binary representation.

The base 10 and hexadecimal numbers would require approximately the same electronic storage space as the binary numbers, if stored as unsigned integers, because these numbers are represented electronically in binary form. UBNIN-C would require much more space than binary because of its large number of base 10 digits.

One advantage of UBNIN-C over other conceivable methods for adjacency matrix encoding is that, with their paper, the authors have provided an unambiguous procedure for how to encode and decode a UBNIN-C. Other methods would also require some sort of extra error-checking information, similar to a checksum number when verifying that a large file has been correctly downloaded [3]. Checksums are routinely employed in verifying the validity of neuroimaging data [4]. However, decoding a UBNIN-C into an adjacency matrix necessarily requires extra information concerning how many columns the adjacency matrix contains, which determines the correct power of 2 for multiplication. Conversion from base 10, with suppression of leading zeros, also requires this information. Binary and hexadecimal data, assuming leading zeros are kept, would not require knowledge of the number of columns because the length of their numbers would indicate the size of the adjacency matrix. However, for the decoding in any form to make sense, the mapping of the columns to their corresponding nodes would also need to be known. Table 2 summarizes potential pros and cons for the use of UBNIN-C compared with binary, base 10, and hexadecimal representations. With the exception of built-in error detection, UBNIN-C does not appear to offer advantages over the other methods. Furthermore, this error detection does not include the integer portion of the UBNIN, which is dropped in the first step of the decoding process and therefore does not affect the final decimal portion for the check (=0, as shown in the right-hand flowchart of Figure 2). Some additional error checking would be needed to include the entire UBNIN-C.

Interestingly, the UBNINs shown in the authors’ abstract only have an integer part, which can only mean that the information for all of the nodes except the last one (column 56) has been discarded and cannot be retrieved by decoding those UBNINs. This is the truncated, UBNIN-T form. The authors explain that “for a larger 20 × 20 fully connected binary matrix, … The UBNIN values hence obtained are rounded up … which is a computational constraint.” By focusing on only the connections for a single node, the number of possible unique combinations of edges, assuming the binary edge values are independent of each other, would drop from 2^1540^ to the more manageable 2^55^, requiring at most 17 digits for UBNIN-T.

It is not clear exactly what the authors meant by “rounding”, but assuming that no data corruption occurs, UBNIN-T is equivalent to a base 10 representation of the last column of the adjacency matrix, so no further analysis is required to understand its properties. The authors apparently attempted to minimize the space needed for these UBNIN-Ts by maximizing the number of leading zeros, which would not be written for these base 10 numbers. They chose to read the numbers from the adjacency matrix from bottom to top because they found that their UBNINs were smaller that way: “The top-to-bottom approach for this methodology was also attempted for multiple adjacency matrices; however, the UBNIN thus obtained was larger than that from bottom-to-top.” Toward this end, it is worth noting that reordering the sequence of the nodes could greatly promote leading zeros. For example, if the node with the greatest number of zero-edges were chosen as the last column, and the other nodes were ordered to position those zeros at the bottom of the column, then leading zeros would be maximized, resulting in minimized base 10 representations of that column.

It is interesting to note that the authors’ “UBNIN_T=10_” value of 321.005979848895 is not the actual UBNIN value that would result from the UBNIN method as described. A comparison with the UBNIN-C value for 10 nodes from Table 1 shows that the authors’ UBNIN_T=10_ value has been rounded to 15 digits, which may be due to the limitations of floating-point calculations using double precision. Spreadsheet programs such as Microsoft Excel and LibreOffice Calc, for example, limit numbers to 15 digits [5]. Decoding 321.005979848895 yields the correct base 10 numbers for each column of the adjacency matrix, with the exception of the second column result of 1.02474780672 rather than exactly 1. Thus, by forgoing UBNIN-C’s error-checking capability, it may be possible to round off a UBNIN-C value to save space, provided that the number of digits remaining exceeds *d* = *n*(*n −* 1)log_10_(2)/2 [2], where *n* is the number of nodes. Some additional questions concerning the processing of such rounded UBNIN values would have to be worked out, however, such as when to round up or down and how close to *d* we can round such values while maintaining the accuracy of the decoded values for the adjacency matrix. The details of such an analysis lie beyond the scope of the current paper.

What matrix size would be ideal for each UBNIN variant? If, for convenience, we use Microsoft Excel or another program that limits numbers to 15 digits, then UBNIN-C would be limited to 6 nodes, because 7 nodes would require a UBNIN-C spanning 17 digits (Table 1). UBNIN-R would not work with more than 10 nodes because 11 nodes would require 17 digits for base 10 representation (exceeding *d* = *n*(*n −* 1)log_10_(2)/2), resulting in loss of information from the original binary matrix with UBNIN-R when rounding to 15 digits. UBNIN-T, limited to 15 base 10 digits, would allow up to 49 nodes (2^49^ distinct binary numbers) by only providing information for a single node, and a higher number of nodes would require a switch to a program that can process numbers longer than 15 digits.

Finally, the word “unique” in the name of the authors’ method requires clarification. The number of possible combinations of edges corresponding to 56 nodes is so large that it seems extremely unlikely that any two individuals would have the exact same adjacency matrix or even the same last column, but technically, their UBNINs are not guaranteed to differ. Even MRI scans for the same person would very likely differ due to scanner drift [6], so in this sense, a UBNIN would be unique for each scan, not for each person. Binary data, if displayed in the original matrix form, retain information concerning connections between nodes. However, information concerning the connections between nodes is not apparent in a UBNIN, so until it is decoded, its primary value would be as a form of storage.

In summary, Samantaray et al. [1] have proposed a new method for representing binary connectivity data, the Unique Brain Network Identification Number. To assist the imaging community in choosing the best method for storage of such data for their needs, we provide a comparison with data stored in binary, base 10, and hexadecimal forms. The authors’ method has three variants: UBNIN-C for small adjacency matrices, UBNIN-R for medium-sized matrices, and UBNIN-T for the largest ones. The advantages of UBNIN-C are that the authors provide a detailed procedure for its encoding and decoding, and it comes with built-in error-checking capability. Disadvantages are that it takes more space in print and in electronic storage than base 10 and hexadecimal representations, and in its encoded form, UBNIN-C no longer provides detailed information concerning the connections between nodes. UBNIN-R allows a more compact representation of matrices, at the cost of losing the error-checking capability, and it can never be more compact than a base 10 representation. For large adjacency matrices, the UBNIN-T method essentially defaults to a base 10 representation of a single node only, which can provide a more compact representation by discarding data from the other nodes and suppressing leading zeros. The uniqueness of adjacency matrices expressed in any UBNIN form is not guaranteed, any more than it would be if expressed in binary, base 10, or hexadecimal forms; however, given the huge number of possible combinations of edges corresponding to 56 nodes, as in the authors’ example, it seems reasonable to assume that no two MRI scans would result in the same UBNIN.

## Figures and Tables

**Figure 1:**
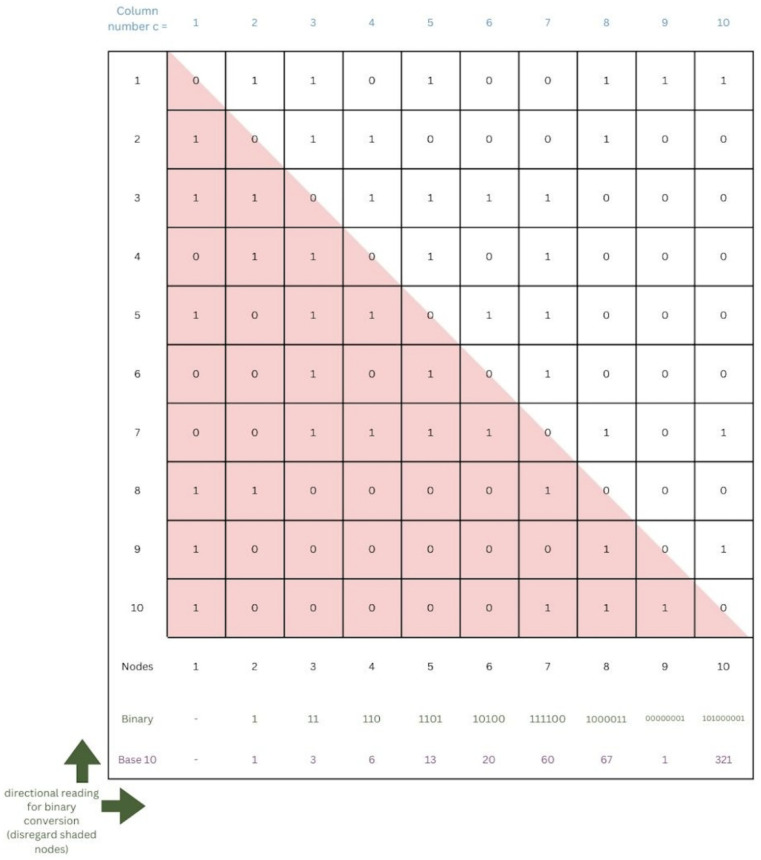
The adjacency matrix example from Samantary et al. [1], showing the lower triangular matrix in pink, in addition to the symmetrical upper triangular matrix that was used to generate the UBNIN. Arrows show the direction chosen by the authors for reading the numbers, bottom-to-top, followed by left-to-right. At the bottom are the base 10 representations for each column. We apply the same reading direction for the construction of our binary, base 10, and hexadecimal numbers.

**Figure 2:**
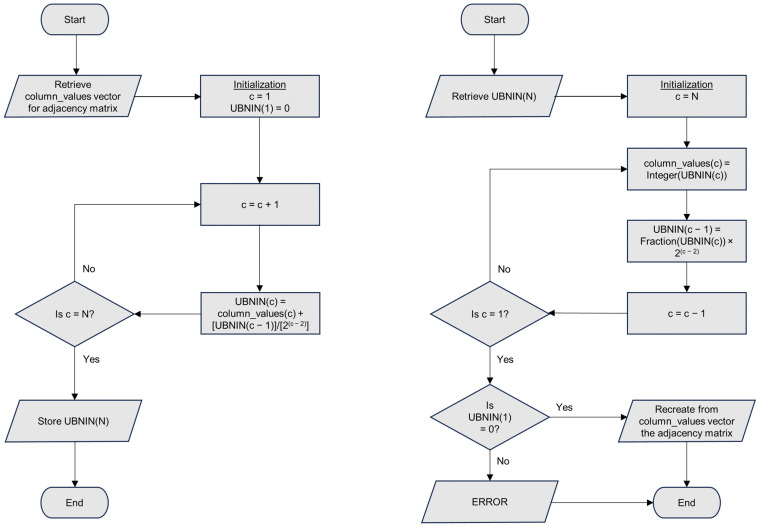
Flowcharts for encoding (**left**) and decoding (**right**) a UBNIN-C for complete representation of a brain-state adjacency matrix corresponding to the connections among *N* nodes. UBNIN(c) gives the UBNIN-C corresponding to the matrix from columns 2 to c, and column_values(c) gives the base 10 value for column c as depicted in Figure 1. Integer(x) gives the integer part of x, and Fraction(x) gives its fractional part. If a UBNIN-C has been successfully decoded, UBNIN(1), the fractional part of UBNIN(2), will always be zero.

**Table 1:** A comparison of the publication space and electronic storage space required to record the brain state for 2–10 nodes, using numbers provided by the authors in their illustrative example of generating the Unique Brain Network Identification Number.

Number of Nodes	UBNIN ^a^			Binary ^b^			Base 10 ^c^			Hexadecimal ^d^		
	Number ^e^	Digits ^f^	Bits ^g^	Number	Digits	Bits	Number	Digits	Bits	Number	Digits	Bits
2	1	1	1	1	1	1	1	1	1	8	1	4
3	3.5	2	6	111	3	3	7	1	3	E	1	4
4	6.875	4	13	111110	6	6	62	2	6	F8	2	8
5	13.859375	8	24	1111101101	10	10	1005	4	10	FB4	3	12
6	20.8662109375	12	38	111110110110100	15	15	32180	5	15	FB6A	4	16
7	60.652069091796875	17	56	111110110110100111100	21	21	2059580	7	21	FB6BE0	6	24
8	67.947688579559326171875	23	76	1111101101101001111001000011	28	28	263626307	9	28	FB6BE43	7	28
9	1.5308413170278072357177734375	29	94	111110110110111111100100001100000001	36	36	67488334593	11	36	FB6BE4301	9	36
10	321.005979848894639872014522552490234375	39	128	111110110110100111100100001100000001101000001	45	45	34554027311937	14	45	FB6BE4301A08	12	48

^a^ UBNIN-C, the UBNIN form that allows reconstruction of the original binary matrix. ^b^ Binary representation, keeping leading zeros. ^c^ Base 10, suppressing leading zeros. ^d^ Hexadeximal, keeping leading zeros, and adding terminal zeros as needed for the last hexadecimal digit. ^e^ The encoded binary matrix in numeric form. ^f^ Number of digits in the encoded number. ^g^ Minimum number of bits required for computer storage, disregarding extra bits for UBNIN decimal point location.

**Table 2:** A comparison of properties for the complete brain-state representations discussed ^a^.

Property	UBNIN-C ^b^	Binary ^c^	Base 10 ^d^	Hexadecimal ^e^
Built-in error detection	Yes	No	No	No
Can show brain connections in print	No	Yes	No	No
Minimizes print space	No	Possibly ^f^	No	Yes
Minimizes electronic storage	No	Yes	Yes	Yes
Uncomplicated decoding back to 1’s and 0’s	No	Yes	No	Yes
Number of nodes required for decoding ^g^	Yes	No	Yes	No
Column-node mapping required ^h^	Yes	Yes	Yes	Yes

^a^ Omitting the abbreviated variants, UBNIN-R and UBNIN-T. ^b^ UBNIN-C is the number generated by the algorithm published by the authors. ^c^ Binary representation of the adjacency matrix, created by concatenating together the binary values for each node. ^d^ Base 10 representation of the adjacency matrix, created by converting the binary representation to base 10. ^e^ Hexadeximal representation of the adjacency matrix, created by converting the binary representation to hexadeximal, padding with zeros at the end as needed for the last hexadeximal digit. ^f^ Binary data can be compressed in print by representing them as light and dark squares, which could require fewer pixels on paper than digits. ^g^ Representations that do not drop leading zeros unambiguously recreate the adjacency matrix without prior knowledge of how many nodes were involved. ^h^ All representations require knowledge of how the nodes were ordered in the adjacency matrix in order to meaningfully map the encoded data back to the original data derived from the brain.

## Data Availability

The primary source of all data presented in the current paper is Samantaray et al.’s article, whose adjacency matrix is shown in Figure 1. The numbers in Table 1 were calculated by hand and entered directly into the table, following the procedures described in the current paper. Matlab R2023b with Symbolic Math Toolbox was used for numbers containing more than 15 digits, and Microsoft Excel 2016 or a hand calculator otherwise. No human data were used for this study.
